# An anoikis-related gene signature predicts prognosis and reveals immune infiltration in hepatocellular carcinoma

**DOI:** 10.3389/fonc.2023.1158605

**Published:** 2023-04-27

**Authors:** Yang Chen, Qiao-xin Lin, Yi-ting Xu, Fang-jing Qian, Chen-jing Lin, Wen-ya Zhao, Jing-ren Huang, Ling Tian, Dian-na Gu

**Affiliations:** ^1^ Department of Clinical Medicine, Wenzhou Medical University, Wenzhou, China; ^2^ Department of Medical Oncology, The First Affiliated Hospital of Wenzhou Medical University, Wenzhou, China; ^3^ Department of Central Laboratory, Shanghai Chest Hospital, Shanghai Jiao Tong University School of Medicine, Shanghai, China

**Keywords:** anoikis, gene signature, hepatocellular carcinoma, prognostic model, immune infiltration

## Abstract

**Background:**

Hepatocellular carcinoma (HCC) is a global health burden with poor prognosis. Anoikis, a novel programmed cell death, has a close interaction with metastasis and progression of cancer. In this study, we aimed to construct a novel bioinformatics model for evaluating the prognosis of HCC based on anoikis-related gene signatures as well as exploring the potential mechanisms.

**Materials and methods:**

We downloaded the RNA expression profiles and clinical data of liver hepatocellular carcinoma from TCGA database, ICGC database and GEO database. DEG analysis was performed using TCGA and verified in the GEO database. The anoikis-related risk score was developed *via* univariate Cox regression, LASSO Cox regression and multivariate Cox regression, which was then used to categorize patients into high- and low-risk groups. Then GO and KEGG enrichment analyses were performed to investigate the function between the two groups. CIBERSORT was used for determining the fractions of 22 immune cell types, while the ssGSEA analyses was used to estimate the differential immune cell infiltrations and related pathways. The “pRRophetic” R package was applied to predict the sensitivity of administering chemotherapeutic and targeted drugs.

**Results:**

A total of 49 anoikis-related DEGs in HCC were detected and 3 genes (EZH2, KIF18A and NQO1) were selected out to build a prognostic model. Furthermore, GO and KEGG functional enrichment analyses indicated that the difference in overall survival between risk groups was closely related to cell cycle pathway. Notably, further analyses found the frequency of tumor mutations, immune infiltration level and expression of immune checkpoints were significantly different between the two risk groups, and the results of the immunotherapy cohort showed that patients in the high-risk group have a better immune response. Additionally, the high-risk group was found to have higher sensitivity to 5-fluorouracil, doxorubicin and gemcitabine.

**Conclusion:**

The novel signature of 3 anoikis-related genes (EZH2, KIF18A and NQO1) can predict the prognosis of patients with HCC, and provide a revealing insight into personalized treatments in HCC.

## Introduction

Hepatocellular carcinoma (HCC), the most prevalent type of liver cancer, is the fourth leading causes of cancer-related death in the world (1). Owing to untypical early symptoms and highly heterogeneous nature, most HCC patients are diagnosed at later stage and lose the opportunity for radical surgery (2). Despite new treatment methods for HCC, such as radiofrequency ablation (RFA), transcatheter arterial chemoembolization (TACE), tyrosine kinase inhibitors (TKIs) and immunotherapy, the prognosis of advanced HCC still remains poor (3). Alarmingly, globally HCC mortality is expected to go up steeply to 41% by 2040 (4). As far, stage-based clinical practice is insufficient for the demands of precision medicine, it is indispensable to identify novel prognostic models for HCC and assist the doctors to choose suitable targets for personalized therapy.

Metastasis is the major cause of death from HCC. Notably, anoikis, a specific form of cell apoptosis, was first described in epithelial and endothelial cells and was found to play a vital role in cancer invasion and metastasis (5, 6). It occurs when cells lose attachment to extracellular matrix (ECM), or adhere to an inappropriate type of ECM, acting as physiological barrier to metastasis (7). Anoikis-resistance is a critical culprit in the metastasis and progression of cancer. In the past decade, we have observed an increasing research progression in the area of tumor anoikis-resistance. Ye et al. found that nuclear MYH9 conferred anoikis resistance to gastric cancer cells and promoted gastric cancer cell metastasis by identifying the CTNNB1 promoter (8). And Wang et al. revealed that CPT1A-mediated fatty acid oxidation could promote colorectal cancer cell metastasis by inhibiting anoikis (9). In addition, a study from UK showed that overexpression of ERBB4 would promote resistance to anoikis and confer enhanced metastatic capacity in Ewing sarcoma (10).

Delineation of novel factors that mitigate anoikis-resistance will open a new avenue for designing therapeutic alternative to trigger cancer cell death and extended survival time. Of note, prognostic model based on the genes related to anoikis had already been established in endometrial carcinoma, glioblastoma and head and neck squamous cell carcinoma, which all displayed excellent predictive ability (11–13). However, few studies have systematically evaluated the link between the anoikis-related genes and the prognosis of HCC patients. Hence, we analyzed the signature of the anoikis-related genes in HCC by using TCGA and ICGC database and constructed a novel prognostic model, and further elucidated the biological functions and immunity-related to the model.

## Materials and methods

### Data acquisition

Gene expression data and corresponding clinical information for patients with liver hepatocellular carcinoma (LIHC) were downloaded from The Cancer Genome Atlas (TCGA) database and The International Cancer Genome Consortium (ICGC) database. The TCGA-LIHC data were used as the training cohort, while those from ICGC were served as the external validation cohorts. Samples with follow-up less than 30 days in TCGA-LIHC data were excluded in this study. In total, 342 patients with HCC were enrolled in the training cohort and 243 patients in the external validation cohort. Transcriptome data were normalized based on the Fragments Per Kilobase of exon model per Million mapped fragments (FPKM). Another set of RNA sequencing data including 268 HCC tumor samples and 243 adjacent non-tumor samples were obtained from Gene Expression Omnibus (GEO) data portal (GSE25097). Similarly, the RNA-seq expression data and corresponding clinical information were also downloaded from the GEO data portal (GSE14520) for additional external validation.

### Identification of anoikis-related DEGs

The anoikis-related genes (ARGs) were extracted from GeneCards (14), and a total of 496 genes were selected with a relevance score >0.4. The “DESeq2” R package was utilized to screen differentially expressed genes (DEGs) (|log2(fold change)| >1 & adjusted p value <0.05) between tumor tissue and tumor adjacent tissue in TCGA count data (15). Then GEO2R was utilized to screen DEGs (|log2(fold change)| >1 & adjusted p-value <0.05) in GSE25097 (16).

### Functional exploration of DEGs

Gene Ontology (GO) and Kyoto Encyclopedia of Genes and Genomes (KEGG) analyses were conducted using the “clusterProfiler” R package to further observe the pathways and functions of anoikis-related genes (17).

### Establishment and validation of risk score model

The univariate Cox regression analysis was used to screen ARGs and genes with a P value<0.05 were considered statistically significant. Then the Least absolute shrinkage and selection operator (LASSO) Cox regression was performed by using the “glmnet” R package to prevent overfitting and construct a gene signature (18). Finally, the multivariate Cox regression analysis was performed to identify strongly correlated genes and build the prognostic gene signature. The risk scores were calculated using the following equation: risk score=∑gene Cox coefficient × gene expression. The median value of risk score was utilized to divide the patients into the high-risk and low-risk group. To evaluate the predictive sensitivity of the model, the Kaplan–Meier (KM) survival curve and the time-dependent receiver operating characteristic (ROC) curves were drawn *via* the “Survival ROC” R package.

### Nomogram construction

All independent prognostic factors were used to construct a prognostic nomogram by the “rms” and “survival” R package. The 1-, 2-, 3-, 5-, and 10-year survival probability for patients with HCC could exactly be predicted by total points, sum points of every factor. Calibrate curves and C-Index values were plotted to estimate the reliability of the survival prediction.

### Functional enrichment analysis

In the low-risk and high-risk groups, DEGs (|log2(fold change)| >1.5 and adjusted p value <0.05) were screened using the “limma” R package. Then, Gene set enrichment analysis (GSEA) was performed *via* the “clusterProfiler” R package to explore signaling pathways (19). Subsequently, the protein-protein interaction (PPI) network for the overlapping DEGs was performed in the STRING database (https://string-db.org/). PPI network interactions file with medium confidence scores ≥ 0.4 was downloaded. We used the open-source software Cytoscape (v 3.9.1) to build PPI network view and to screen out hub genes in the DEGs. According to the median value of hub genes’ expression, HCC patients in the TCGA and ICGC were categorized into low- and high- group to further explore prognosis relevance.

### Immune infiltrate analysis

The ESTIMATE (Estimation of STromal and Immune cells in Malignant Tumour tissues using Expression data) algorithm was performed to calculate the stromal score, immune score, tumor purity and ESTIMATE score between high-risk and low-risk groups (20). Immune score and stromal score were employed to assess the immune cell infiltration and the presence of stroma in the TME, and the sum of the stromal and immune scores was evaluated by ESTIMATE score. The cell-type identification by estimating relative subsets of RNA transcripts (CIBERSORT) analysis was used to evaluate the relative abundance of 22 immune cell types between them (21). The statistical significance of the deconvolution results was assessed to filter out the samples with less significant accuracy by a derived P-value (P < 0.05). And the infiltrating scores of 16 immune cells and 13 immune-related pathways were calculated by applying the single-sample gene set enrichment analysis (ssGSEA) method from the “GSVA” R package (22). The “c2.cp.kegg.v7.4.symbols” files were downloaded from the MSigDB database for GSVA analysis. Moreover, Pearson correlation analysis was utilized to explore the association between risk score and the expression of the immune checkpoint genes, such as PD-1, PD-L1 and CTLA-4. P value < 0.05 was considered statistically significant.

### Drug sensitivity analysis

The “pRRophetic” R package was used to calculate the half-maximal inhibitory concentration (IC50) values of chemotherapeutic and targeted drugs for each HCC sample (23). Moreover, transcriptome data and clinical data of the IMvigor210 immunotherapy cohort (bladder cancer) were obtained using the “IMvigor210CoreBiologies” R package.

### Mutation analysis

We downloaded the mutation data of TCGA-LIHC patients from TCGA database. The “maftools” R package was used to assess the mutation profile between the low-risk and high-risk group (24). We also calculated the tumor mutation burden (TMB) score for every HCC patient (25).

### TISCH2

TISCH2 (Tumor Immune Single-cell Hub 2) is a scRNA-seq database (http://tisch.comp-genomics.org/), which aims to characterize tumor microenvironment (TME) at single-cell resolution (26). In this study, we used TISCH2 database to decipher expression of the 3 anoikis-related genes in TME of hepatocellular carcinoma.

### Statistical analysis

DEGs were screened using the Wilcoxon test. Box plot analyses were performed using the Wilcoxon rank-sum test. We used the K-M curve to do univariate survival analyses, with comparison by log-rank test. P value < 0.05 was defined as statistically significant. Statistical analyses were done by R version 4.2.1 (R Foundation for Statistical Computing, Vienna, Austria).

## Results

**Figure 1:**
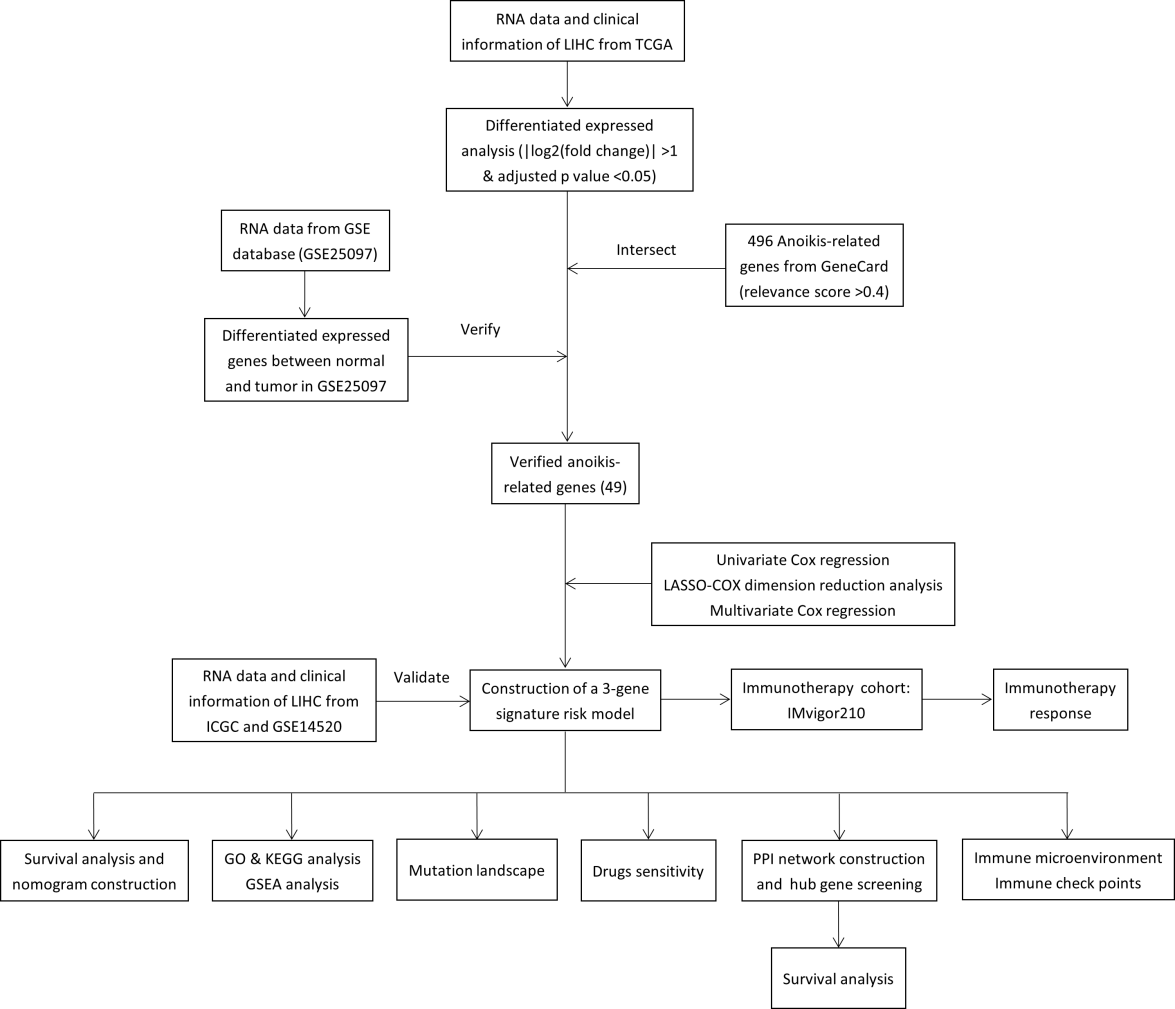
Flow chart of the study.

Totals of 342 and 243 patients with HCC from TCGA and ICGC data were selected as the training and external validation group, respectively. The flowchart of the study is shown in **Figure 1**.

### Determination of anoikis-related DEGs

**Figure 2:**
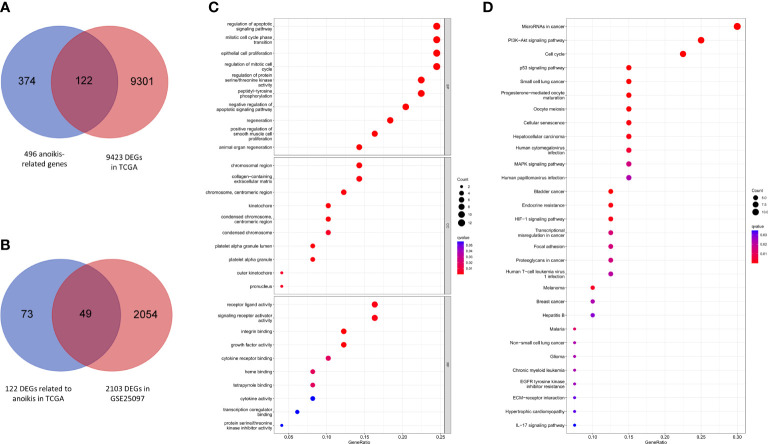
Anoikis-related gene screening and functional analysis. **(A)** Anoikis-related DEGs in TCGA. **(B)** Further validation in GSE25097. **(C)** GO enrichment analysis of 49 anoikis-related DEGs in HCC. **(D)** KEGG pathways analysis of 49 anoikis-related DEGs in HCC.

A total of 9423 DEGs were collected after analysis of the gene expression data of HCC samples and corresponding control tissues in TCGA cohort. Then these DEGs were intersected with the anoikis-related gene set to get 122 ARGs in TCGA (**Figure 2A**). Next, the ARGs in TCGA were verified with the DEGs in GSE25097. Finally, 49 DEGs related to anoikis were determined for further analysis (**Figure 2B**).

### Functional analysis of anoikis-related genes in HCC

According to the results of GO functional analysis, we found that the most highly enriched biological processes of 49 ARGs were regulation of apoptotic signaling pathway, mitotic cell cycle phase transition and epithelial cell proliferation (**Figure 2C**). KEGG pathway analysis showed that the microRNAs in cancer, PI3K-Akt signaling pathway and cell cycle were mainly enriched (**Figure 2D**). These findings indicated the potential molecular mechanisms involved in the regulation of HCC progression by anoikis-related DEGs.

### Construction and validation of anoikis-related prognosis signature

**Figure 3:**
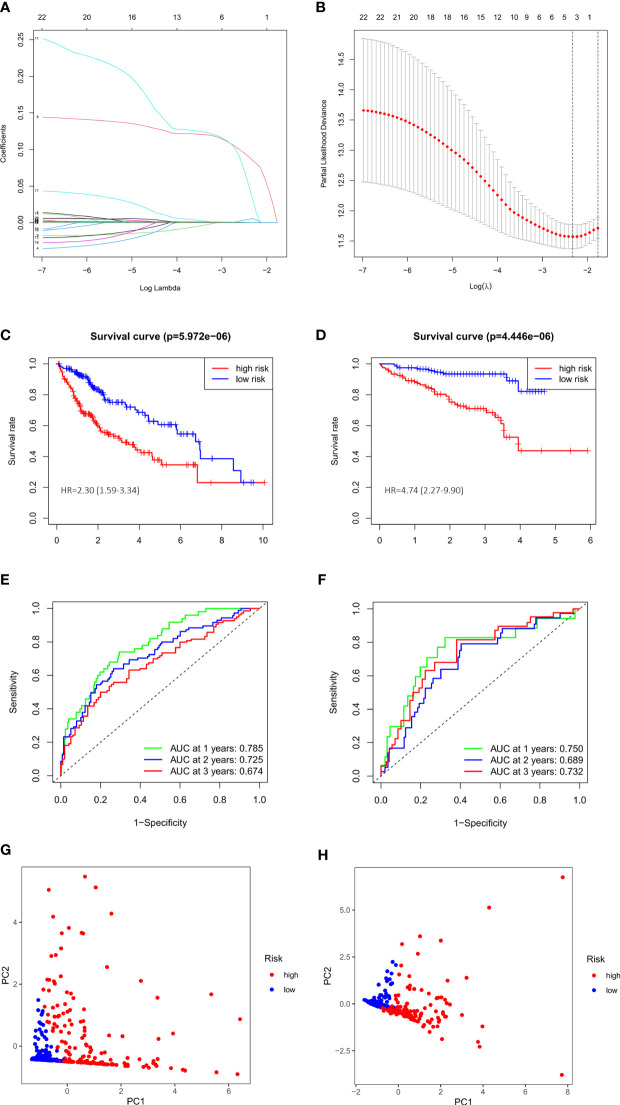
Evaluation and validation of 3-gene signature in TCGA cohort and ICGC cohort. **(A)** LASSO coefficient profiles of 23 prognostic genes of HCC. **(B)** LASSO regression with tenfold cross-validation found 4 prognostic genes using the minimum λ. **(C)** Kaplan–Meier curves for OS in TCGA cohort. **(D)** Kaplan–Meier curves for OS in ICGC cohort. **(E)** Time-dependent ROC curves for OS in TCGA cohort. **(F)** Time-dependent ROC curves for OS in ICGC cohort. **(G)** PCA plot of risk score in TCGA cohort. **(H)** PCA plot of risk score in ICGC cohort.

We performed a univariate Cox regression and found that 23 of DEGs have potential prognostic significance (P<0.05). Then, a LASSO logistic regression analyses was conducted to further screen 4 key anoikis-related prognostic genes (**Figures 3A, B**). 3 genes (EZH2, KIF18A, NQO1) were finally identified by multivariate Cox regression to build a prognostic model as follows: risk score= (EZH2 × 0.141741668) + (KIF18A × 0.190725435) + (NQO1 × 0.001887712). Subsequently, 342 patients from TCGA-LIHC were divided into low- and high-risk group according to the median value of risk score. PCA analysis showed that the two groups could be well-distinguished by the ARGs (**Figure 3G**). Then the K-M curve showed that the high-risk group had worse clinical outcome than that of low-risk group (HR=2.30, 95% CI =1.59-3.34) (**Figure 3C**). The area under the time-dependent ROC curves (AUCs) for 1‐, 2‐ and 3‐year were 0.785, 0.725, 0.674, respectively, demonstrating a favorable prediction performance of the prognostic model (**Figure 3E**). To ensure the prediction value of the identified anoikis-related prognosis signature, 171 HCC patients were randomly selected from the TCGA-LIHC cohort as the testing set. Similar to the results obtained from the training set, the constructed model exhibited great performance for OS prediction (**Supplementary Figures S1A-C-E**).

We also validated the prediction performance of gene signature in independent external validation set. According to the same formula, 243 patients with HCC from ICGC data were used to verify this prognostic model. As the same results in TCGA, K-M curve revealed that the patients in the high-risk group had a significantly worse OS than their low-risk counterparts (HR=4.74, 95% CI =2.27-9.90) (**Figure 3D**). PCA analysis further confirmed two remarkably different risk groups (**Figure 3H**). And the 1-, 2- and 3-year AUCs of the training group were 0.750, 0.689, 0.732, respectively (**Figure 3F**), which indicated the predictive reliability of model in both training and validation group. Consistent with the results in the TCGA training cohort and the ICGC external validation group, we observed similar trends in another external validation group GSE14520 (**Supplementary Figures S1B–F**). Moreover, we plotted heatmaps of expression difference in the 3 independent prognostic genes and displayed the impact of risk scores on risk ranking, survival time and survival status in TCGA training group and ICGC external validation group (**Supplementary Figure S2**). Meanwhile, we explored the protein expression of the 3 genes in the Human Protein Atlas and found them all strongly stained in HCC specimens compared with normal liver specimens (**Supplementary Figure S3**). The results further verified that the signature had significant prognostic value for HCC patients.

### Creating predictive nomograms

**Figure 4:**
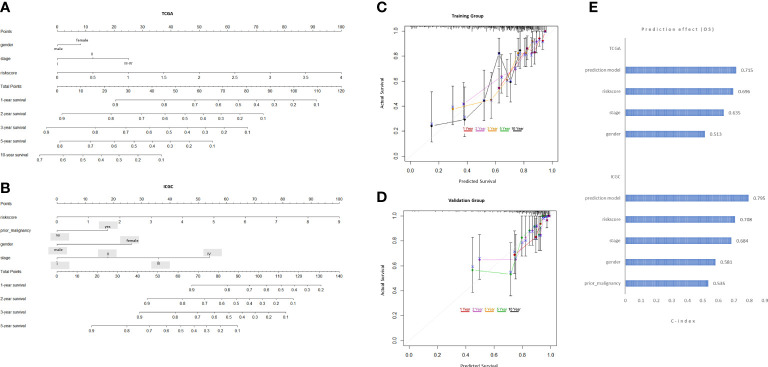
Development and validation of the predictive nomogram. **(A, B)** The nomogram construction based on the TCGA cohort and ICGC cohort. **(C, D)** The Calibration plot of the nomogram in training and validation groups. **(E)** C-index values of the nomogram.

To expand the application of the prognostic model, individualized nomograms were constructed. In the training group, gender, stage and risk score were selected in the final model (**Figure 4A**). In the validation group, gender, stage, prior malignancy and risk score were chosen in the final model (**Figure 4B**). The C‐index values for prediction model were 0.715, 0.795 in the training group and validation group, respectively (**Figure 4E**). The nomograms displayed excellent predictive ability for OS for HCC patients. Moreover, the calibration curve showed good uniformity in both training and validation model, indicating an appropriate predictive accuracy (**Figures 4C, D**).

### Functional analysis

**Figure 5:**
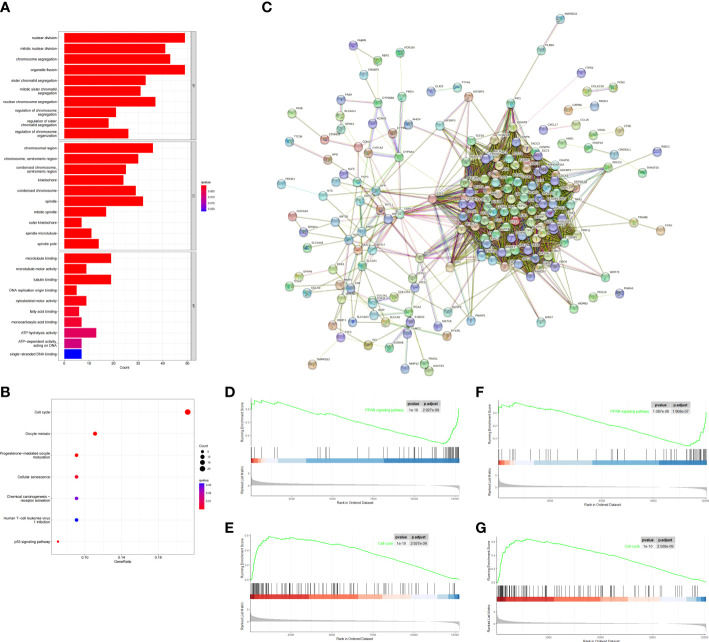
Functional analysis based on the DEGs between the two-risk groups in the TCGA and ICGC cohort. **(A)** Barplot graph for GO enrichment (the longer bar means the more genes enriched, and the increasing depth of red means the differences were more obvious; q-value: the adjusted p value). **(B)** Bubble graph for KEGG pathways. The larger bubble means the more genes enriched, and the increasing depth of red means the differences were more obvious. q-value, the adjusted p value. **(C)** PPI network of 229 DEGs. **(D–G)** Two representative Kyoto Encyclopedia of Genes and Genomes (KEGG) pathways *via* GSEA in TCGA cohort **(D, E)**, ICGC cohort **(F, G)**.

To investigate biological process between the two risk groups, we obtained the DEGs *via* the “limma” R package from both training and validation group. Then we intersected them and finally obtained 229 overlapping DEGs (**Figure 5C**). Primary information of 229 DEGs was summarized in **Supplementary Table 1**. The GO function enrichment analyses showed that the DEGs were mainly associated with nuclear division, mitotic nuclear division and chromosome segregation (**Figure 5A**). The KEGG enrichment analyses revealed that the DEGs were enriched in cell cycle, oocyte meiosis and cellular senescence (**Figure 5B**). GSEA analyses showed that cell cycle signaling pathway was enriched in the high-risk group, while PPAR signaling pathway was enriched in the low-risk group (**Figures 5D–G**). The results may help to explain why high-risk group had worse overall survival.

### Exploration of TIME

**Figure 6:**
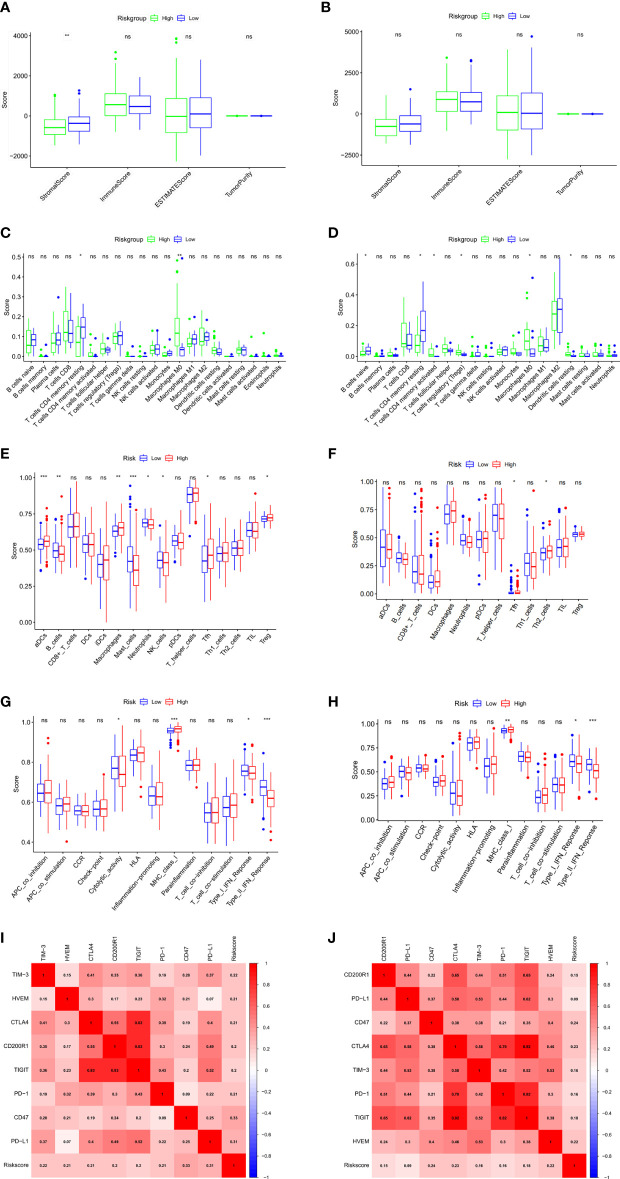
Evaluation of tumor microenvironment and immune checkpoints of anoikis-related prognostic signature. **(A, B)** Comparison between the estimate score, immune score, stromal score, and tumor purity based on risk groups in TCGA cohort **(A)** and ICGC cohort **(B)**. **(C, D)** Comparison between the subtypes of immune cells based on risk groups in TCGA cohort **(C)** and ICGC cohort **(D)**. **(E, F)** Immune cell infiltration analysis based on risk groups in TCGA cohort **(E)** and ICGC cohort **(F)**. **(G, H)** Immune-related pathways infiltration analysis based on risk groups in TCGA cohort **(G)** and ICGC cohort **(H)**. **(I, J)** The correlations between the risk score and the expression of the immune checkpoint genes in TCGA cohort **(I)** and ICGC cohort **(J)**. *p < 0.05; **p < 0.01; ***p < 0.001; ns, not significant.

To explore the correlation of anoikis-related gene signature with immunotherapy, we next performed ESTIMATE algorithm to compare the difference in tumor immune microenvironment (TIME) between two groups. The results displayed that the stromal score tended to decrease in the high-risk group although not significant in the ICGC validation cohort (**Figures 6A, B**). By GSVA analysis, we found that the expression of KEGG immune pathways linked with complement and coagulation cascades was lower, while the expression of IL−17 signaling pathway was higher in that high-risk group compared with the low-risk group (**Supplementary Figure S1G**). Regarding 22 types of TIICs in HCC from the CIBERSORT algorithm, we observed significantly higher proportions of M0 macrophages and lower proportions of T cells CD4 memory resting in the high-risk group (**Figures 6C, D**). Besides, the results of ssGSEA algorithm showed the high-risk group gained lower ssGSEA score in the immune pathways, including the type I IFN response and type II IFN response (**Figures 6E–H**). These data indicated that high-risk group may contribute to tumor immune dysfunction in HCC. As for Pearson correlation analysis of immune checkpoints, the heat map showed that the expression of immune checkpoints, especially CD47, was found to have a positive correlation with risk score (**Figures 6I, J**). In short, these results suggested that the anoikis-related gene signatures might affect the efficacy of immunotherapy in HCC patients.

### Evaluation efficacy of cancer therapeutic agents in different risk groups

**Figure 7:**
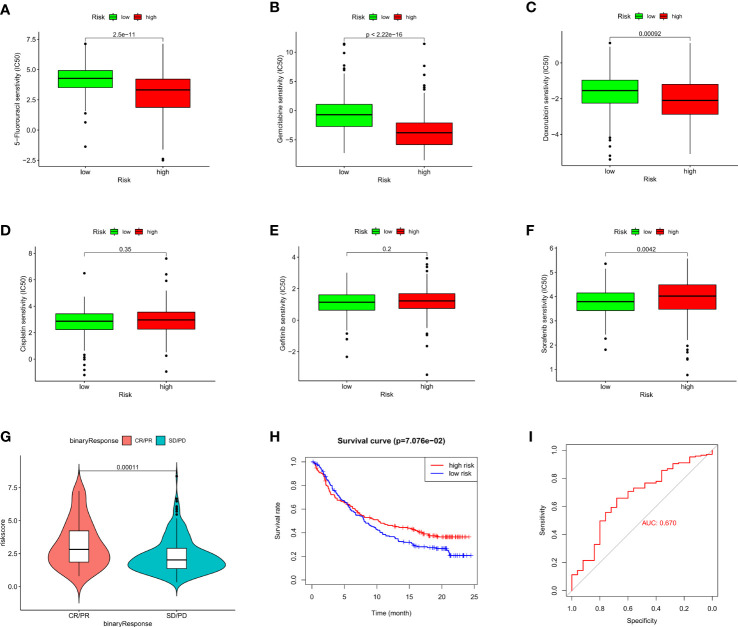
Evaluation of chemosensitivity and immunotherapeutic responses to PD-L1 by the risk model. **(A–F)** Therapeutic efficacy of 5-Fluorouracil **(A)**, Gemcitabine **(B)**, Doxorubicin **(C)**, Cisplatin **(D)**, Gefitinib **(E)**, and Sorafenib **(F)** in different risk groups. **(G)** The comparison of risk score between SD/PD and CR/PR two groups in IMvigor210 cohort. **(H)** Kaplan–Meier curves for OS in IMvigor210 cohort. **(I)** ROC curves of risk score in predicting the immunotherapy response.

To assess the therapeutic efficacy of chemotherapeutic and targeted drugs for HCC in low-risk and high-risk group, we used the “pRRophetic” R package to calculate the half inhibitory concentration (IC50) of six commonly used drugs (5-fluorouracil, cisplatin, doxorubicin, gemcitabine, gefitinib, and sorafenib) for treating HCC (**Figure 7**). The analysis showed that sorafenib had a high drug response to the low-risk group (**Figure 7F**). In contrast, 5-fluorouracil, doxorubicin and gemcitabine were observed to present a significant response to high-risk group (**Figures 7A–C**). Moreover, to verify whether the prognostic model we built could effectively predict the efficacy of immunotherapy, we applied the IMvigor210 database as an external anti-PD-L1 cohort. We discovered that patients in complete response/partial response (CR/PR) group had higher risk scores compared with those in stable disease/progressive disease (SD/PD) group (**Figure 7G**). And the ROC curve depicted that the risk score model displayed a great predictive effect to ICIs response (**Figure 7I**). These findings demonstrated that grouping based on the ARGs could promote individualized therapy for HCC patients, and patients in the high-risk group may be more likely to benefit from immunotherapy.

### The relationship between anoikis-related gene signature and mutation profile in HCC

**Figure 8:**
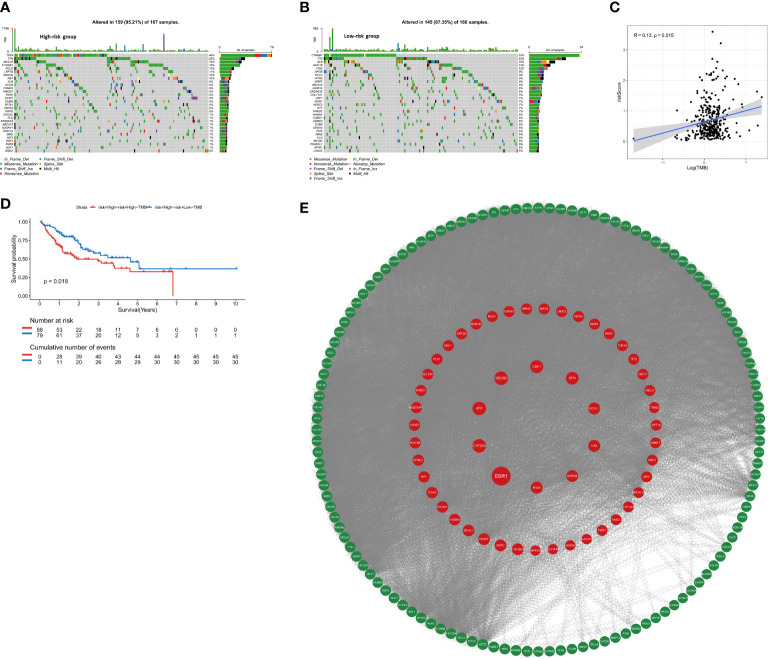
The mutation profile and TMB among low-risk and high-risk groups. **(A)** Mutation profile in the high-risk group. **(B)** Mutation profile in the low-risk group. **(C)** The relationship between the anoikis-related risk signature and TMB. **(D)** Kaplan–Meier curves for HCC patients stratified by TMB in high-risk group. **(E)** PPI network construction and top ten hub genes screening.

To assess whether the mutation profile differed between the high-risk and low-risk group, the somatic mutation data of 333 HCC patients from TCGA was used for analysis. We exhibited the top 30 mutated genes in two risk groups, the gene with the highest mutation frequency is TP53 (46%) in the high-risk group and that in the low-risk group is CTNNB1 (33%) (**Figures 8A, B**). Besides, more mutations were discovered in patients in the high-risk group compared with those in the low-risk group. As shown in **Figure 8C**, there was no significant correlation between riskScore and TMB (r=0.13, p=0.015). For further comparative studies, the high-risk group was divided into the high-risk&Low-TMB subgroup and high-risk&high-TMB subgroup based on the median value of TMB. Subsequently, the K-M curve showed that the high-risk group exhibited significant prognostic difference in the high and the low TMB value subgroups (P=0.018, **Figure 8D**). These findings might contribute novel insight into the intrinsic connection between the individual somatic mutations and the anoikis-related gene signature.

### PPI network construction and hub genes screening

The STRING database was utilized to construct an interaction network between proteins encoded by the 229 DEGs (**Figure 8E**). ANLN, NDC80, AFP, ESR1, CA9, UCHL1, CYP3A4, CDK1, SFN, AURKB were identified as the top 10 hub genes. Detailed information on 10 genes was listed in **Supplementary Table 2**. Depending on the median expression of the 10 genes, patients were grouped into low- and high-expression groups to further explore the differences in survival. The K-M curve analysis showed that high-expression groups of ANLN, AURKB, CDK1, NDC80 were consistent with shorter OS, while CYP3A4 and ESR1 demonstrated opposite results (**Supplementary Figures S4A–L**). To further investigate whether these 6 hub genes were clinically independent prognostic factors, we performed multivariate COX regression and found that ANLN was an independent prognostic gene in both training and validation cohorts (**Supplementary Figures S4M, N**). Subsequently Pearson correlation analysis showed ANLN exhibited a significantly positive correlation with the expression of the immune checkpoint gene CD47 (**Supplementary Figure S5C**). Additionally, from the GSCALite website (http://bioinfo.life.hust.edu.cn/web/GSCALite/), we found that patients with high expression of ANLN were less likely to benefit from multiple drugs, such as 5-FU and Methotrexate (**Supplementary Figure S5A**).

### Correlation between the three anoikis-related genes and the tumor microenvironment of HCC

**Figure 9:**
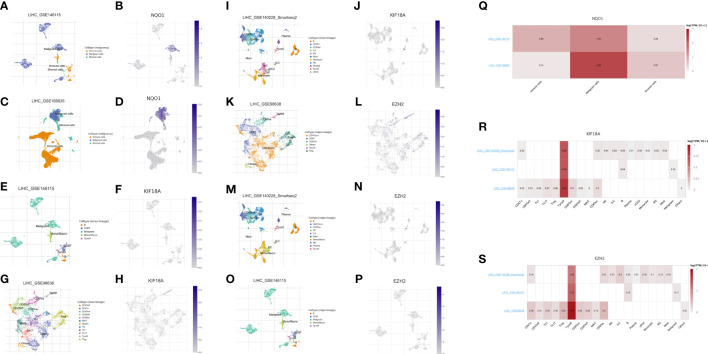
Expression of 3 anoikis-related genes in LIHC TME-associated cells. **(A, C)** The cell types and their distribution in LIHC_GSE146115 and LIHC_GSE166635 datasets. **(B, D)** Distribution of NQO1 in different cells in LIHC_GSE146115 and LIHC_GSE166635 datasets. **(E, G, I)** The cell types and their distribution in LIHC_GSE146115, LIHC_GSE98638 and LIHC_GSE140228 datasets. **(F, H, J)** Distribution of KIF18A in different cells in LIHC_GSE146115, LIHC_GSE98638 and LIHC_GSE140228 datasets. **(K, M, O)** The cell types and their distribution in LIHC GSE98638, LIHC_GSE140228 and LIHC_GSE146115 datasets. **(L, N, P)** Distribution of EZH2 in different cells in LIHC GSE98638, LIHC_GSE140228 and LIHC_GSE146115 datasets. **(Q)** Expressions of NQO1 in LIHC_GSE146115 and LIHC_GSE166635 datasets. **(R)** Expressions of KIF18A in LIHC_GSE146115, LIHC_GSE98638 and LIHC_GSE140228 datasets. **(S)** Expressions of EZH2 in LIHC GSE98638, LIHC_GSE140228 and LIHC_GSE146115 datasets.

We evaluated the expression of the three anoikis-related genes (NQO1, KIF18A, EZH2) in the four single-cell sequencing HCC datasets of GSE146115, GSE166635, GSE98638, GSE140228 datasets from TISCH2 database. The distribution and number of various cell types of the HCC datasets were visualized in **Figures 9A–P**. The results showed NQO1 was mainly expressed in malignant cells (**Figure 9Q**), while KIF18A and EZH2 were highly expressed in Tprolif cells (**Figures 9R, S**).

## Discussion

HCC is one of the most lethal malignant tumors, with a 5-year survival rate of just 3% (27). Due to the high levels of intratumoral heterogeneity in HCC, effective treatment modalities are still scarce. The incidence and mortality rates of HCC nearly mirror each other. The incidence rate in Eastern Asia of HCC is 17.7 per 100,000 persons, whereas the corresponding mortality rate was 16.0 (28). Therefore, it is still necessary to find biomarkers for predicting prognosis and evaluating therapeutic response to optimize clinical decision-making for HCC patients.

Anoikis is a type of programmed cell death, which is activated when cells are detached from extracellular matrix (ECM) (29). Cancer cells must develop anoikis resistance before forming metastatic foci in distant organs. Also, anoikis tolerance is responsible for the treatment failure of several types of cancer. Therefore, there is an urgent need to explore the anoikis-related genes on invasive mobility and their role in predicting the prognosis of cancer. Along with the rapid development of various types of sequencing technologies, bioinformatics analysis has become an important tool for the research of molecular mechanism in cancer (30, 31). Zhao et al. constructed a 7 anoikis-related genes signature to predict the survival of Low-grade glioma (LGG) patients (32), Chen et al. identified 5 prognostic anoikis-related genes (CHEK2, PDK4, ZNF304, SNAI2, SRC) to establish a risk-predictive model for clear cell renal cell carcinoma (33), which all exhibited great predictive performance and implemented as a stratification factor for individualized treatment. Moreover, Sun et al. also revealed the potential relationships between anoikis-related genes and glioblastoma (13). Similarly, anoikis also plays a critical role in HCC metastasis. Therefore, the anoikis-related model may also be utilized to predict the prognosis of HCC patients. To our best knowledge, limited research has been conducted on the establishment of prognostic model using anoikis-related gene in HCC.

In our study, we proposed robust 3-gene signature, namely EZH2, KIF18A and NQO1. EZH2 plays a vital role in cell cycle progression (34), DNA damage repair and cellular senescence (35), and regulates relative signaling pathways in cell lineage determination (36). A study from Greece found EZH2 is regulated by ERK/AKT and targets integrin alpha2 gene to control epithelial–mesenchymal transition (EMT) and anoikis in colon cancer cells (37). Recently, Lei et al. demonstrated that circSYPL1 promotes the proliferation and metastasis of HCC *via* the upregulation of EZH2 expression by competing with hsa-miR-506-3p (38). As for KIF18A, it is a member of the kinesin superfamily and works as a master regulator of chromosome aggregation and centromere movements. In a previous study, KIF18A has been identified as a potential therapeutic target for human breast cancer (39). Luo et al. also suggested that KIF18A may promote proliferation, invasion and metastasis of HCC cells by promoting the cell cycle signaling pathway, the Akt signaling pathway and the MMP-7/MMP-9-related signaling pathways (40). NQO1 is a gene that encodes a cytoplasmic 2-electron reductase. Researchers have found that NQO1 is associated with aging and early pathological changes in Alzheimer’s disease (AD) (41, 42). Moreover, Shimokawa et al. reported that modulation of NQO1 activity could intercept anoikis resistance and suppresses HCC metastasis (43), while Yang et al. drew a conclusion that NQO1 could promote an aggressive phenotype in hepatocellular carcinoma *via* enhancing ERK-NRF2 signaling (44). From our work, after analysis of 4 HCC scRNA-seq datasets, we found that two of the 3-gene signature, KIF18A and EZH2 had correlation coefficient with Tprolif cells, which indicated KIF18A/EZH2/Tprolif cells axis might be a pathway in initiation and progression of HCC. And NQO1 was mainly expressed in malignant cells. In summary, these three genes play a vital role in anoikis and are closely related to the prognosis of HCC.

Moreover, we established a nomogram for clinical-decision support. Nomogram, a visual statistical tool, was wildly used in prognostic prediction of cancer patients (45). In the current study, combining the 3-anoikis gene signature, gender, prior malignancy and TNM stage, a prognosis nomogram with excellent performance was constructed. The C-index of the nomograms constructed based on TCGA and ICGC database were 0.715, 0.795, respectively. Since these independent prognostic factors included in nomogram construction are easy to obtain from the clinical practice, the nomogram may be used routinely in the future.

Immunotherapy has attracted worldwide attention for its anti-cancer activity (46). In the past decade, the immunotherapeutics targeting PD-1/PD-L1 and CTLA-4 has achieved gratifying results on HCC patients (47, 48). However, only a limited number of HCC patients benefited from it, the efficacy of immunotherapy is affected by many factors specific to the individual, such as the unique TIME, the expression of the immune checkpoint genes and the related gene mutation levels. Tumor immune microenvironment (TME) has a significant impact on tumor progression process and therapy response, so that many researchers would conduct immune cell infiltration and TME analysis of tumors of their interest (49–51). In our work, with the application of CIBERSORT algorithm and ssGSEA approach, we found that macrophage M0 infiltration was greater in the high-risk group, while T cells CD4 memory resting was less infiltration. And the high-risk group had decreased infiltration in the immune pathways, including the type I IFN response and type II IFN response. These findings suggested that the higher immunosuppression and lower immunoreactivity in TME may account for the worse prognosis for high-risk patients with HCC. Moreover, we found that the expression of the majority of immune checkpoint genes was positively correlated with the risk score. Taken together, these results suggested that the anoikis-related gene signatures might affect the efficacy of immunotherapy in HCC patients and the model could be identified as an immunotherapy indicator.

To further establish the relationship between the anoikis-related model and ICI therapy, we included 348 patients with urothelial cancer from IMvigor210 cohort for further analysis. We observed that there was statistically significant difference in the overall survival between high-risk and low-risk groups. As well, patients in complete response/partial response (CR/PR) group had higher risk scores than those in stable disease/progressive disease (SD/PD) group. Moreover, given that chemotherapy is still the gold standard for cancer treatment, we also evaluated the effectiveness of the anoikis-related model in distinguishing chemotherapy outcomes. In our study, we found the high-risk group had high sensitivity to 5-fluorouracil, doxorubicin and gemcitabine, while the low-risk group was more sensitive to sorafenib. The above results verified the anoikis-related model may aid in the development of individualized treatment of HCC patients.

Needless to say, the study still has some limitations. First, the study relied solely on publicly available datasets, which might bring selection bias, the real-world prospective cohort studies would be needed to validate the results. Second, due to the shortage of public data on HCC patients receiving anti-PD-L1 antibody, we used the IMvigor210 cohort (bladder cancer) as external immunotherapy cohort. Meanwhile, the underlying mechanisms of ARGs needed further experimental verification.

In conclusion, our study constructed a novel anoikis-related 3-gene signature (EZH2, KIF18A and NQO1), which exhibited favorable prediction performance. We also assessed the differences in immunotherapy response and chemotherapeutic drug sensitivity between the two risk groups, thereby providing a new insight for clinical treatment.

## Data availability statement

The original contributions presented in the study are included in the article/**Supplementary Material**. Further inquiries can be directed to the corresponding authors.

## Ethics statement

Ethical review and approval was not required for the study on human participants in accordance with the local legislation and institutional requirements. Written informed consent for participation was not required for this study in accordance with the national legislation and the institutional requirements.

## Author contributions

YC and Q-xL: data analysis, formal analysis, and writing - original draft; Y-tX and F-jQ: visualization and data curation; C-jL, W-yZ, and J-rH: supervision and data collection; LT and D-nG: funding acquisition, resources, supervision, and writing - review and editing. All authors contributed to the article and approved the submitted version.

## Supplementary material

Supplementary Figure 1 The risk model performance. **(A)** Kaplan–Meier curves for OS in TCGA testing cohort. **(B)** Kaplan–Meier curves for OS in GSE14520 cohort. **(C)** Time-dependent ROC curves for OS in TCGA testing cohort. **(D)** Time-dependent ROC curves for OS in GSE14520 cohort. **(E)** PCA plot of risk score in TCGA testing cohort. **(F)** PCA plot of risk score in GSE14520 cohort. **(G)** Heatmap illustrating the result of GSVA.

Supplementary Figure 2 Risk score distribution, survival status and heatmap of expression profiles in low- and high-risk groups.

Supplementary Figure 3 Immunohistochemical images obtained from the Human Protein Atlas.

Supplementary Figure 4 Survival analysis of 10 hub genes from 229 DEGs. **(A–L)** Kaplan–Meier curves of 6 hub genes in TCGA cohort **(A–F)**, ICGC cohort **(G–L)**. **(M, N)** Multivariate Cox regression analysis was carried out for the OS of 6 hub genes in TCGA cohort **(M)** and ICGC cohort **(N)**.

Supplementary Figure 5 Evaluation of chemosensitivity and the mutation level of hub genes from 229 DEGs.
