# Supplementary figures and images for: An anoikis-related gene signature predicts prognosis and reveals immune infiltration in hepatocellular carcinoma
Source: Front Oncol. 2023 Apr 27;13:1158605. doi: 10.3389/fonc.2023.1158605 (PMC10172511; doi:10.3389/fonc.2023.1158605)

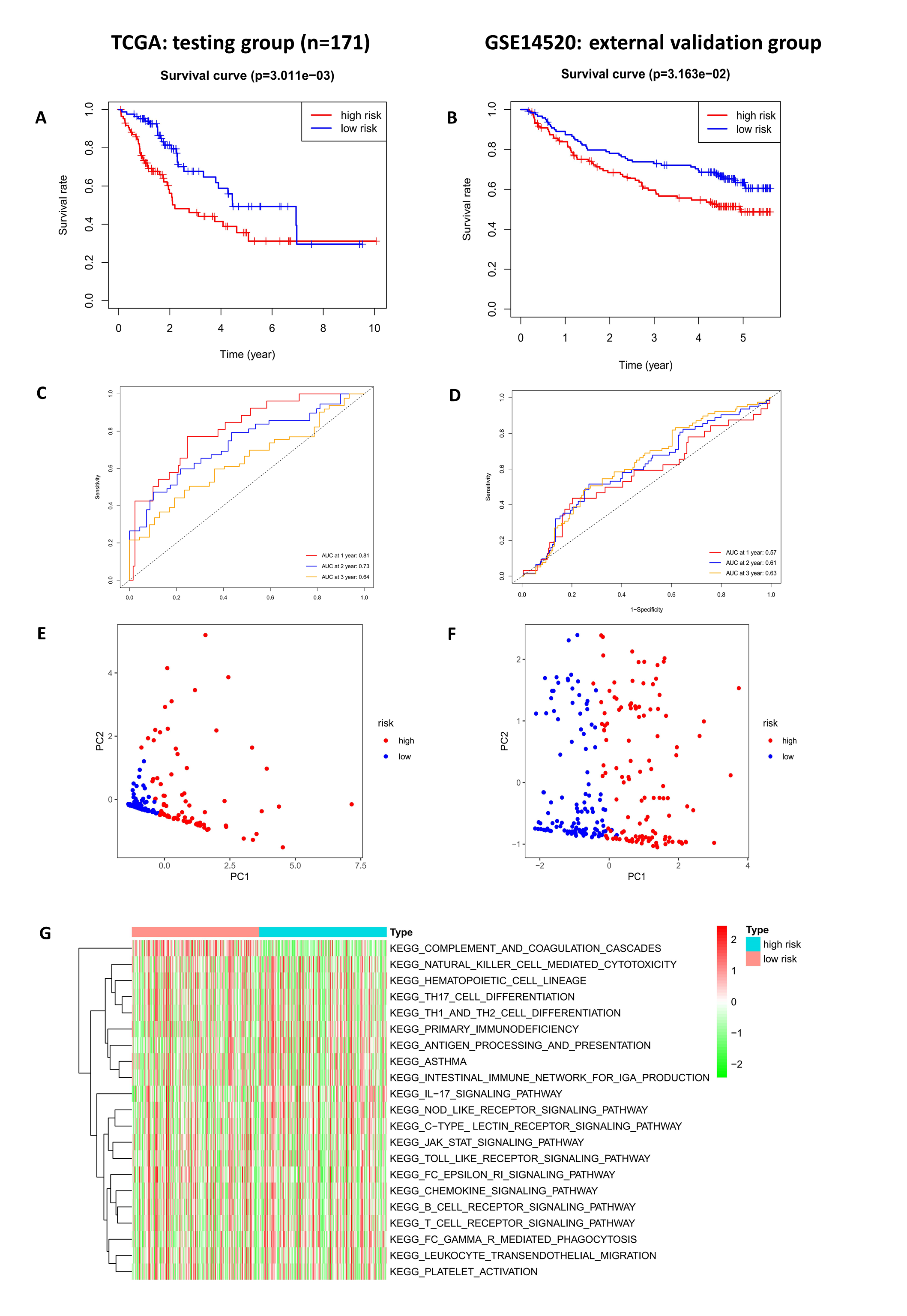

Supplement: Supplementary Figure 1 — The risk model performance. (A) Kaplan–Meier curves for OS in TCGA testing cohort. (B) Kaplan–Meier curves for OS in GSE14520 cohort. (C) Time-dependent ROC curves for OS in TCGA testing cohort. (D) Time-dependent ROC curves for OS in GSE14520 cohort. (E) PCA plot of risk score in TCGA testing cohort. (F) PCA plot of risk score in GSE14520 cohort. (G) Heatmap illustrating the result of GSVA. [file Image_1.tif]

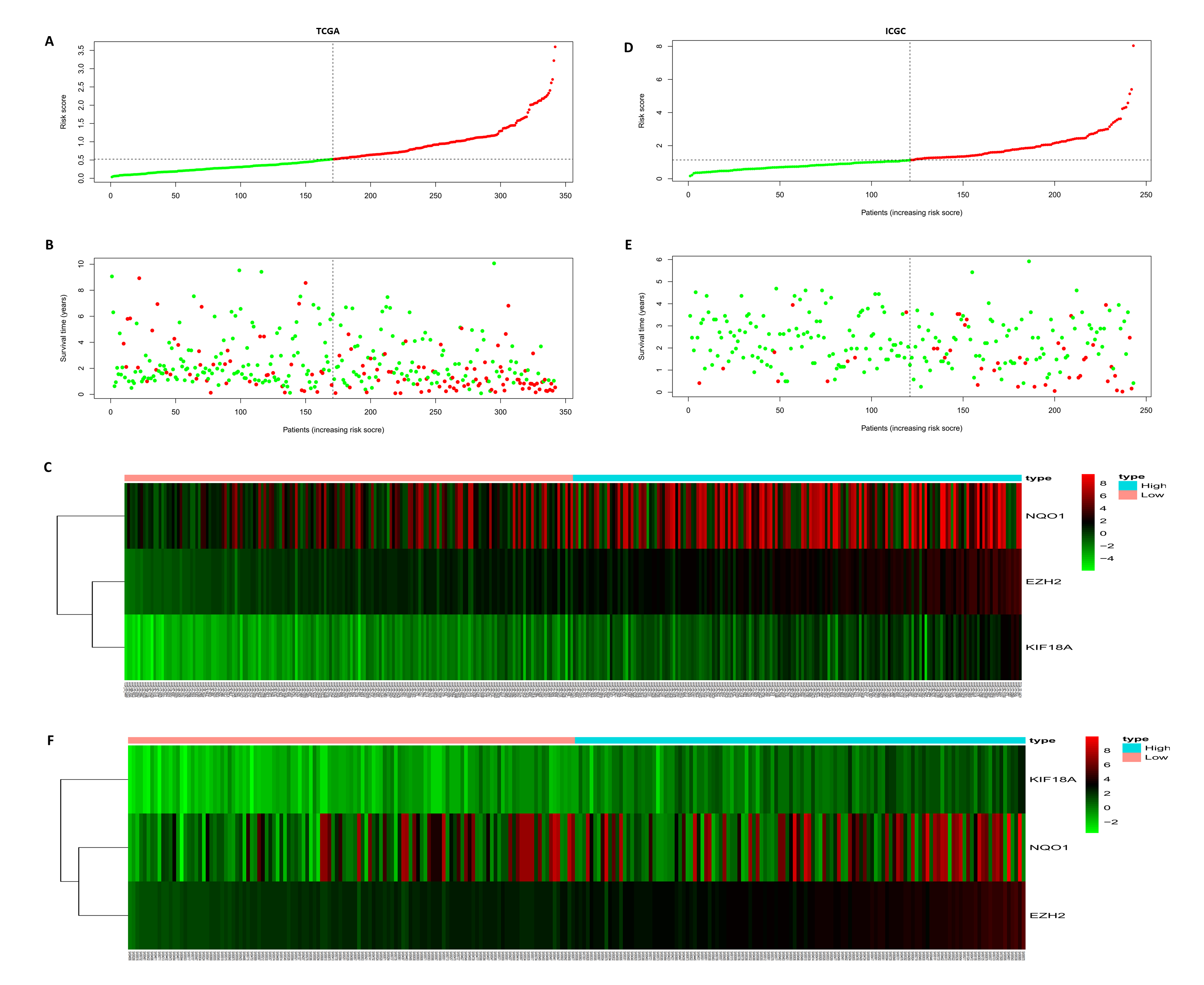

Supplement: Supplementary Figure 2 — Risk score distribution, survival status and heatmap of expression profiles in low- and high-risk groups. [file Image_2.tif]

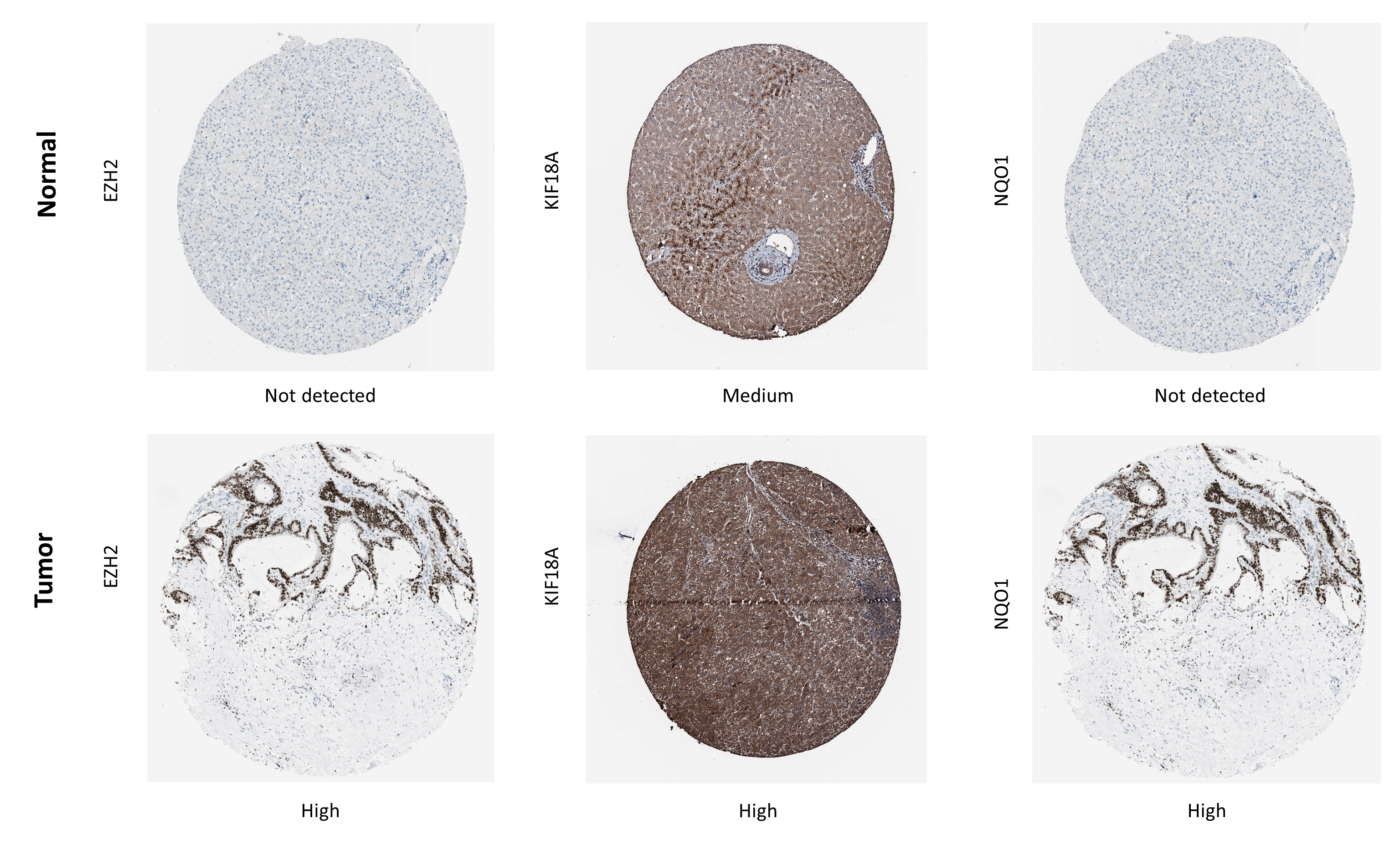

Supplement: Supplementary Figure 3 — Immunohistochemical images obtained from the Human Protein Atlas. [file Image_3.tif]

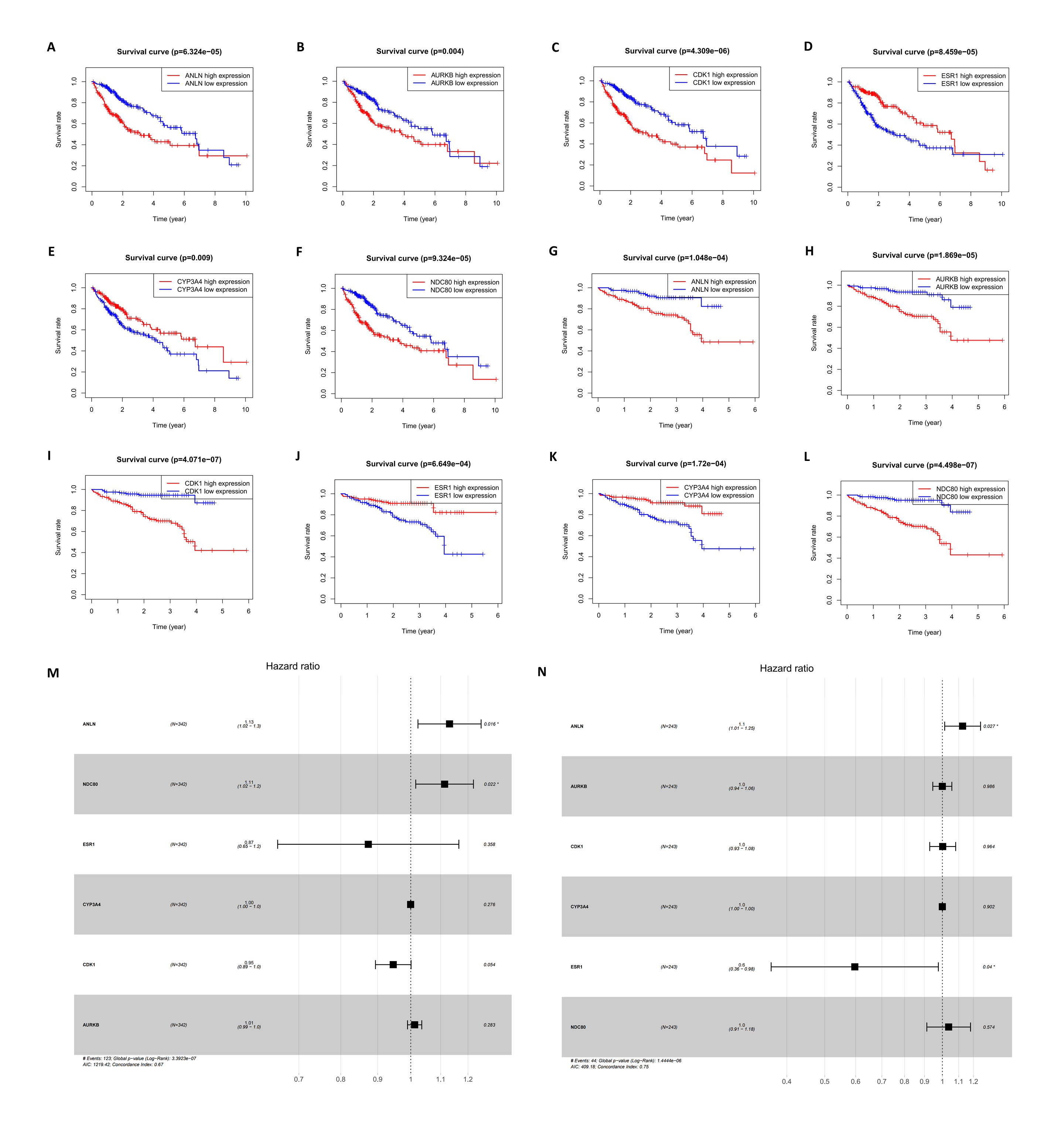

Supplement: Supplementary Figure 4 — Survival analysis of 10 hub genes from 229 DEGs. (A–L) Kaplan–Meier curves of 6 hub genes in TCGA cohort (A–F), ICGC cohort (G–L). (M, N) Multivariate Cox regression analysis was carried out for the OS of 6 hub genes in TCGA cohort (M) and ICGC cohort (N). [file Image_4.tif]

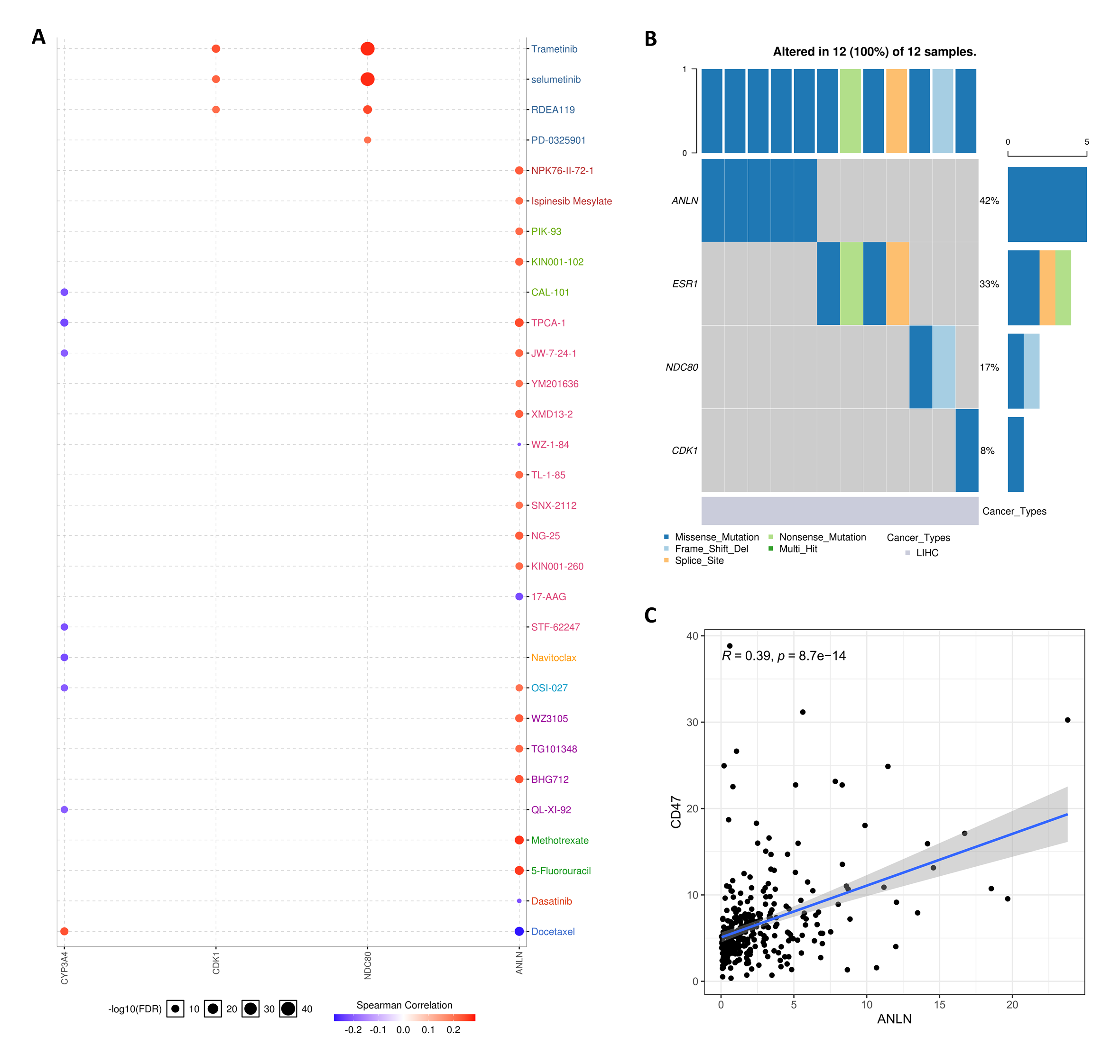

Supplement: Supplementary Figure 5 — Evaluation of chemosensitivity and the mutation level of hub genes from 229 DEGs. [file Image_5.tif]
