# Supplementary material for: An anoikis-related gene signature predicts prognosis and reveals immune infiltration in hepatocellular carcinoma
Source: Front Oncol. 2023 Apr 27;13:1158605. doi: 10.3389/fonc.2023.1158605 (PMC10172511; doi:10.3389/fonc.2023.1158605)
Supplement: Supplementary file 7 [file Table_2.docx]

| **Table 2. Detail information of ten hub genes.** | | |
| --- | --- | --- |
| Gene symbol | Description | Function of the encoded protein |
| ANLN | Anillin, Actin Binding Protein | This gene encodes an actin-binding protein that plays a role in cell growth and migration, and in cytokinesis. |
| NDC80 | NDC80 Kinetochore Complex Component | This protein functions to organize and stabilize microtubule-kinetochore interactions and is required for proper chromosome segregation. |
| AFP | Alpha Fetoprotein | This gene encodes alpha-fetoprotein, a major plasma protein produced by the yolk sac and the liver during fetal life. The level of alpha-fetoprotein in amniotic fluid is used to measure renal loss of protein to screen for spina bifida and anencephaly. |
| ESR1 | Estrogen Receptor 1 | The protein encoded by this gene regulates the transcription of many estrogen-inducible genes that play a role in growth, metabolism, sexual development, gestation, and other reproductive functions and is expressed in many non-reproductive tissues. |
| CA9 | Carbonic Anhydrase 9 | Carbonic anhydrases (CAs) are a large family of zinc metalloenzymes that catalyze the reversible hydration of carbon dioxide. They participate in a variety of biological processes, including respiration, calcification, acid-base balance, bone resorption, and the formation of aqueous humor, cerebrospinal fluid, saliva, and gastric acid. |
| UCHL1 | Ubiquitin C-Terminal Hydrolase L1 | The protein encoded by this gene belongs to the peptidase C12 family. This enzyme is a thiol protease that hydrolyzes a peptide bond at the C-terminal glycine of ubiquitin. |
| CYP3A4 | Cytochrome P450 Family 3 Subfamily A Member 4 | This gene encodes a member of the cytochrome P450 superfamily of enzymes. The cytochrome P450 proteins are monooxygenases that catalyze many reactions involved in drug metabolism and synthesis of cholesterol, steroids and other lipids. |
| CDK1 | Cyclin Dependent Kinase 1 | The protein encoded by this gene is a member of the Ser/Thr protein kinase family. This protein is a catalytic subunit of the highly conserved protein kinase complex known as M-phase promoting factor (MPF), which is essential for G1/S and G2/M phase transitions of eukaryotic cell cycle. |
| SFN | Stratifin | The encoded protein binds to translation and initiation factors and functions as a regulator of mitotic translation. In response to DNA damage this protein plays a role in preventing DNA errors during mitosis. |
| AURKB | Aurora Kinase B | This gene encodes a member of the aurora kinase subfamily of serine/threonine kinases. These kinases participate in the regulation of alignment and segregation of chromosomes during mitosis and meiosis through association with microtubules. |
